# Particulate Metabolites and Transcripts Reflect Diel Oscillations of Microbial Activity in the Surface Ocean

**DOI:** 10.1128/mSystems.00896-20

**Published:** 2021-05-04

**Authors:** Angela K. Boysen, Laura T. Carlson, Bryndan P. Durham, Ryan D. Groussman, Frank O. Aylward, François Ribalet, Katherine R. Heal, Angelicque E. White, Edward F. DeLong, E. Virginia Armbrust, Anitra E. Ingalls

**Affiliations:** a School of Oceanography, University of Washington, Seattle, Washington, USA; b Department of Biology, Genetics Institute, University of Florida, Gainesville, Florida, USA; c Department of Biological Sciences, Virginia Tech, Blacksburg, Virginia, USA; d Daniel K. Inouye Center for Microbial Oceanography: Research and Education (C-MORE), University of Hawaii, Honolulu, Hawaii, USA; Scripps Institution of Oceanography

**Keywords:** metabolomics, North Pacific, phytoplankton, diel cycles, microbial ecology, oceanography, osmolytes, transcriptomics

## Abstract

Light fuels photosynthesis and organic matter production by primary producers in the sunlit ocean. The quantity and quality of the organic matter produced influence community function, yet *in situ* measurements of metabolites, the products of cellular metabolism, over the diel cycle are lacking. We evaluated community-level biochemical consequences of oscillations of light in the North Pacific Subtropical Gyre by quantifying 79 metabolites in particulate organic matter from 15 m every 4 h over 8 days. Total particulate metabolite concentration peaked at dusk and represented up to 2% of total particulate organic carbon (POC). The concentrations of 55/79 (70%) individual metabolites exhibited significant 24-h periodicity, with daily fold changes from 1.6 to 12.8, often greater than those of POC and flow cytometry-resolvable biomass, which ranged from 1.2 to 2.8. Paired metatranscriptome analysis revealed the taxa involved in production and consumption of a subset of metabolites. Primary metabolites involved in anabolism and redox maintenance had significant 24-h periodicity and diverse organisms exhibited diel periodicity in transcript abundance associated with these metabolites. Compounds with osmotic properties displayed the largest oscillations in concentration, implying rapid turnover and supporting prior evidence of functions beyond cell turgor maintenance. The large daily oscillation of trehalose paired with metatranscriptome and culture data showed that trehalose is produced by the nitrogen-fixing cyanobacterium *Crocosphaera*, likely to store energy for nighttime metabolism. Together, paired measurements of particulate metabolites and transcripts resolve strategies that microbes use to manage daily energy and redox oscillations and highlight dynamic metabolites with cryptic roles in marine microbial ecosystems.

**IMPORTANCE** Fueled by light, phytoplankton produce the organic matter that supports ocean ecosystems and carbon sequestration. Ocean change impacts microbial metabolism with repercussions for biogeochemical cycling. As the small molecule products of cellular metabolism, metabolites often change rapidly in response to environmental conditions and form the basis of energy and nutrient management and storage within cells. By pairing measurements of metabolites and gene expression in the stratified surface ocean, we reveal strategies of microbial energy management over the day-night cycle and hypothesize that oscillating metabolites are important substrates for dark respiration by phytoplankton. These high-resolution diel measurements of *in situ* metabolite concentrations form the basis for future work into the specific roles these compounds play in marine microbial communities.

## INTRODUCTION

Marine microorganisms and the organic matter they use and produce form the foundation of marine ecosystems. Though particulate organic carbon (POC) in the surface ocean is primarily macromolecules (1, 2), a suite of small molecules (metabolites less than ∼800 Da) produced within cells helps shape the internal and external chemical environment of the plankton community, creating potential dependencies among different taxa. However, an inventory of these compounds and the plasticity of their concentrations remain largely unknown (3). Measurements of the chemical diversity and concentration of metabolites present in marine microbial communities are scarce, and the suite of compounds detected is strongly biased by the methods employed (4). Small polar molecules, in particular, are rarely measured, although they are the main component of the aqueous cytosol (5). Intracellular metabolite profiles of model marine microbes are taxon-specific and respond to environmental perturbations, including diel oscillations in available light (6). Measurements of many dynamic metabolites have not yet been conducted in natural plankton communities, and some metabolites are without annotated biosynthetic or catabolic pathways (7–13). Thus, an *in situ* inventory of intracellular metabolites will facilitate a deeper understanding of marine microbial physiology and interactions that drive ecosystem diversity and activity (14, 15).

The diel oscillation of light fuels phytoplankton photosynthesis and the organic matter production that supports ocean ecosystems and carbon sequestration (16). In surface waters, the direct or indirect consequences of this diel forcing can be seen in daily oscillations in cell division (17), gross primary production, net community production (18), grazing (19), viral infection (20), and nitrogen fixation (21). Genes associated with a wide variety of cellular processes also exhibit diel oscillations in transcript abundance, reflecting the capture of light energy and its conversion to chemical energy during daylight, a process that fuels metabolism over a 24-h period (22–26). Temporal partitioning of anabolism and catabolism creates diel patterns in total POC and in the macromolecular composition of POC (26–34).

Here, we measured particulate metabolite concentrations in samples collected from surface waters near Station ALOHA (A Long-Term Oligotrophic Habitat Assessment; 22.75° N, 158° W) in the North Pacific Subtropical Gyre (NPSG) during eight daily cycles. These data provide an inventory of metabolites in the oligotrophic surface ocean over the diel cycle. We paired metabolite concentrations with observations of gene expression, POC, particulate nitrogen (PN), and flow cytometry (FCM) cell counts. The measurements of low molecular weight metabolites at the molecular and temporal resolution presented here provide new details about the timing and breadth of synchronous metabolic activities in natural microbial communities in the surface ocean.

We find that the molar concentration of 70% of our targeted metabolites oscillated with 24-h periodicity, reflecting large-scale community synchrony to the daily cycle of light. Our analysis identifies diel oscillations in compounds that play important roles in managing light-induced redox reactions and biosynthesis of building blocks and energy stores. Oscillating concentrations of compounds with osmotic properties confirm prior work suggesting that these compounds, referred to here as osmolytes, can have many alternative cellular functions beyond maintaining cell turgor pressure. The metabolites we measure are ultimately conduits of energy and nutrients through the microbial ecosystem as they are exchanged between diverse organisms, either through active excretion or after passive exudation or cell death. The quantity and quality of the organic matter produced and used within cells thus have repercussions for community diversity and function (35–37). Our metabolite data reveal a dynamic component of the chemical environment within natural populations of marine plankton. Paired with metatranscriptomes, these data point to potential metabolic strategies that organisms deploy to cope with an oscillating energy supply.

## RESULTS

### Oscillatory dynamics of the phytoplankton community.

**FIG 1:**
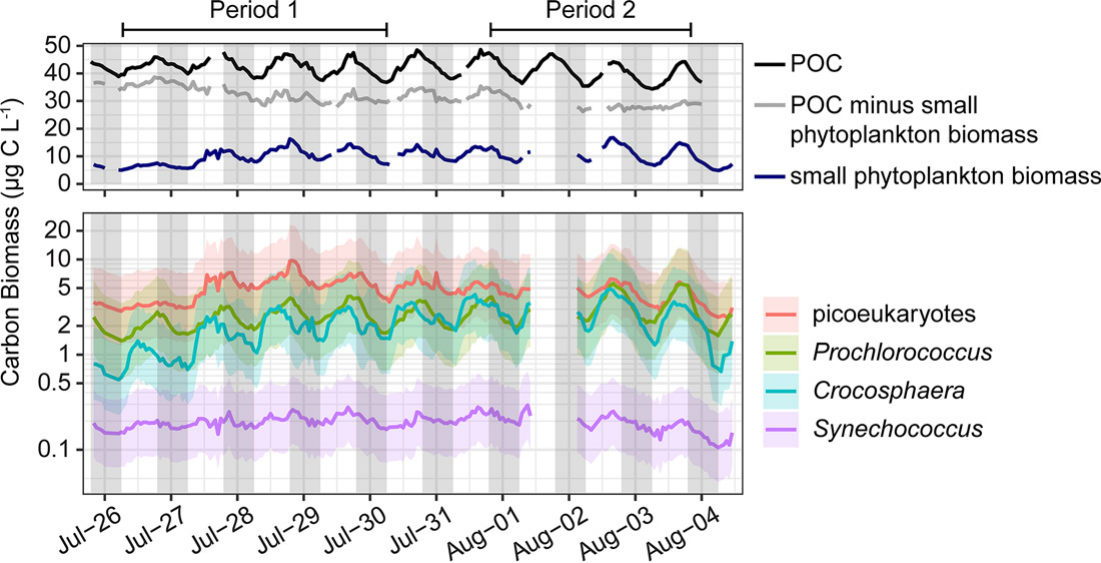
Top: Hourly averages of POC from beam attenuation (black line, RAIN FDR-corrected *P < *0.001), total phytoplankton carbon biomass from flow cytometry (small phytoplankton biomass, navy line, RAIN FDR-corrected *P < *0.001), and the difference between the two (gray line, RAIN FDR-corrected *P > *0.05). Bottom: Hourly averages of population specific carbon biomass of *Prochlorococcus*, *Synechococcus*, *Crocosphaera*, and photosynthetic picoeukaryotes (defined here as 2 to 4 μm) from flow cytometry, with shaded area representing the 95% confidence interval (RAIN FDR-corrected *P < *0.001 for all four populations); note the log_10_-scaled *y* axis. Breaks in the lines are due to short periods of instrument malfunction and removal of large spikes. The two sampling periods referred to in the text are indicated above the figure.

Our sampling targeted an anticyclonic eddy and followed two drifters to facilitate Lagrangian sampling of surface ocean water with minimal mixing or forcing other than the day-night cycle. Samples were collected for two multiday sampling periods in summer 2015 (period one: July 26, 6:00 to July 30, 6:00; period two: July 31, 18:00 to August 3, 18:00). The eddy was characterized by warm, nutrient-deplete surface waters typical of the persistently oligotrophic NPSG (19, 38) (Table 1). Photosynthetic picoeukaryotes and the cyanobacteria *Prochlorococcus* and *Crocosphaera* contributed substantially to phytoplankton biomass (21) (Fig. 1). POC, which includes bulk community biomass, and picophytoplankton-specific biomass oscillated with significant 24-h periodicity (Fig. 1). Cell abundances and total biomass of *Prochlorococcus* and *Crocosphaera* populations increased between the first and second sampling periods (Table 1). Wind speed also increased between the first and second sampling periods, resulting in an increase in the mixed layer depth from 21 ± 5 to 36 ± 6 m. Additionally, we observed a decrease in the number of significantly diel metabolite oscillations during the second sampling interval, from 55 to 9 (see Table S1 in the supplemental information). Here, we focus our analysis on data collected during the first sampling period where metabolite oscillations were more pronounced.

**TABLE 1:** Wind speed and surface mixed layer physical and biological variables over the two sampling periods^*a*^

Characteristic	First sampling period [avg ± SD (*n*)]	Second sampling period [avg ± SD (*n*)]
Wind speed (kts)	10.59 ± 1.97 (83)	15.15 ± 1.76 (72)
Mixed layer depth (m)	20.92 ± 5.20 (25)	36.12 ± 6.42 (19)
Salinity	35.38 ± 0.01 (62)	35.39 ± 0.00 (33)
Temp (°C)	26.81 ± 0.10 (268)	26.86 ± 0.07 (80)
Nitrate + nitrite (nM liter^−1^)	3.02 ± 0.90 (19)	3.34 ± 1.65 (9)
Dissolved oxygen (μmol kg^−1^)	205.74 ± 0.70 (63)	205.62 ± 0.43 (39)
Particulate organic carbon (μmol C liter^−1^)	3.52 ± 0.23 (94)	3.39 ± 0.32 (73)
Particulate nitrogen (μmol N liter^−1^)	0.44 ± 0.04 (18)	0.40 ± 0.03 (12)
Heterotrophic bacteria abundance (10^6^ cells liter^−1^)	508.3 ± 27.3 (22)	534.1 ± 30.8 (47)
*Prochlorococcus* abundance (10^6^ cells liter^−1^)	161.23 ± 11.75 (98)	196.38 ± 15.37 (55)
*Synechococcus* abundance (10^6^ cells liter^−1^)	0.85 ± 0.07 (98)	0.89 ± 0.06 (55)
Photosynthetic picoeukaryote abundance (10^6^ cells liter^−1^)	0.97 ± 0.11 (98)	1.10 ± 0.33 (55)
*Crocosphaera* abundance (10^6^ cells liter^−1^)	0.16 ± 0.06 (98)	0.31 ± 0.07 (55)

a Salinity, temperature, and dissolved oxygen (corrected with bottle measurements) are from the CTD between 13 and 17 m. Nitrate plus nitrite and heterotrophic bacteria abundance are measured from discrete samples at 15 m. Particulate organic carbon (from underway beam attenuation), particulate nitrogen, *Prochlorococcus*, *Synechococcus*, photosynthetic picoeukaryotes, and *Crocosphaera* are measured from the ship-underway water intake near 7 m. SD, standard deviation.

10.1128/mSystems.00896-20.6 TABLE S1 Metabolites measured in this analysis. The average fold change from peak to trough, the maximum and minimum estimated or absolutely quantified values after calculating the median values of the biological triplicates collected at each sampling time point (pmol liter^−1^), and whether the compound oscillates with 24-hour periodicity when calculated as molar concentration in seawater (water), when calculated relative to POC (POC), both (Both), or neither (None) for the first sampling period analyzed independently, second sampling period analyzed independently, and full dataset. The times of peak concentration for these concentration values in the different time periods are provided in the final columns, rounded to the nearest hour. The net flux through the particulate pool calculated by the mean daily swing from max to minimum. An asterisk (*) indicates metabolites for which samples 21 to 24 are removed and for which 6 samples in the second diel sampling period may be affected by internal standard adjustments. A plus sign (+) indicates metabolites for which 4 samples in the second sampling period might be affected by internal standard (IS) adjustments. A dagger symbol (†) notes that concentrations for DMSP are likely underestimates, as described in Materials and Methods. Download Table S1, CSV file, 0.01 MB. Copyright © 2021 Boysen et al. 2021 Boysen et al. https://creativecommons.org/licenses/by/4.0/ This content is distributed under the terms of the Creative Commons Attribution 4.0 International license.

### Metabolite inventory.

**FIG 2:**
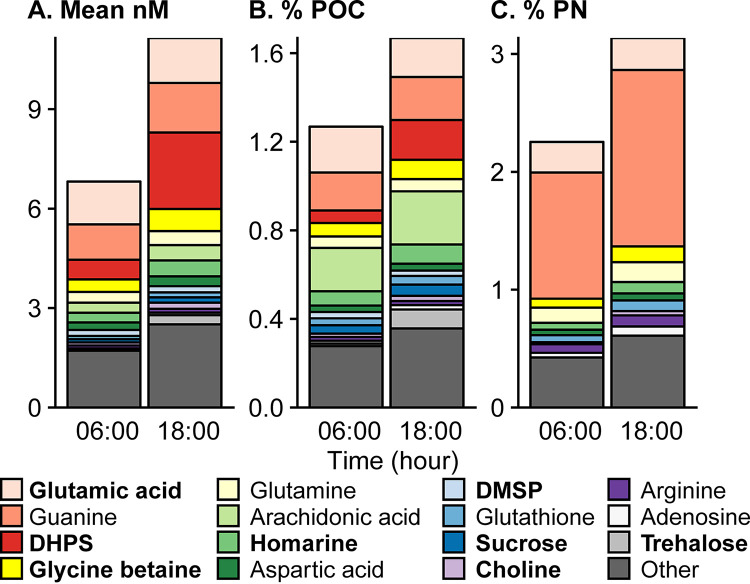
Average targeted metabolite composition at dawn (06:00) and dusk (18:00) from July 26 to July 28 (triplicate samples per time point over 3 days results in *n *=* *9 for each time point), shown as the estimated particulate metabolite concentration (nmol liter^−1^) (A), the percentage of particulate organic carbon (B), and the percentage of the particulate nitrogen (C). “Other” contains the sum of the rest of the metabolites (64 compounds). Compounds with osmotic properties are in bold. Metabolites are arranged according to their average molar concentration at 6:00. Note the different *y* axis scales. Standard deviations for these estimates are in Table S2.

A total of 79 targeted metabolites were detected across samples (Table S1; Data set S1). Total particulate metabolite concentration (as molar concentration and as a percentage of POC or PN) increased during the day and decreased at night (Fig. 2). The most abundant compounds were osmolytes, including glycine betaine (GBT), homarine, 2,3-dihydroxypropane-1-sulfonate (DHPS), and dimethylsulfoniopropionate (DMSP), nucleobases (particularly guanine), and amino acids related to nitrogen metabolism, such as glutamic acid and glutamine (Tables S1 and S2; Fig. 2). At dusk, quantified metabolites totaled 1.7% ± 0.2% of POC and 3.1% ± 0.6% of PN (Fig. 2), with free nucleobases and amino acids comprising most of the metabolite derived nitrogen (2.5% ± 1.1% of PN at dusk, Table S2).

10.1128/mSystems.00896-20.7 TABLE S2 Average and standard deviation of targeted metabolite composition at dawn (06:00) and dusk (18:00) from July 26 to July 28 (*n *=* *9 for each time point), as the estimated particulate metabolite concentration, the percentage of particulate organic carbon, and the percentage of the particulate nitrogen. Download Table S2, CSV file, 0.01 MB. Copyright © 2021 Boysen et al. 2021 Boysen et al. https://creativecommons.org/licenses/by/4.0/ This content is distributed under the terms of the Creative Commons Attribution 4.0 International license.

10.1128/mSystems.00896-20.9 DATA SET S1 Particulate metabolite concentrations in normalized peak area per liter of seawater filtered. Across a single metabolite these values are proportional to molar concentration. Values should not be quantitatively compared between two metabolites, since the ionization efficiency and matrix effects influence different metabolites differently such that the same concentration can result in difference in peak area. Download Data Set S1, XLSX file, 0.1 MB. Copyright © 2021 Boysen et al. 2021 Boysen et al. https://creativecommons.org/licenses/by/4.0/ This content is distributed under the terms of the Creative Commons Attribution 4.0 International license.

Multivariate analyses were used to determine if time of day influenced the community metabolome. NMDS analysis indicated that samples collected at different times had different overall metabolomes (ANOSIM, *R *=* *0.19, *P = *0.001). Samples collected near sunrise (6:00) had metabolomes that were more similar to one another than those of samples collected at other times of day, and the metabolomes collected at 6:00 were most dissimilar to those from samples collected near sunset (Fig. S1; Table S3).

10.1128/mSystems.00896-20.2 FIG S1 Multivariate analyses based on standardized particulate metabolite concentration (proportional to nmol liter^−1^). (A) NMDS of the first sampling period alone: July 26th to July 30th. The NMDS analysis results were significant (Monte Carlo randomization *P < *0.01) with a stress value of 0.18. (B) Within and between group variability from ANOSIM analysis using standardized particulate concentrations of all metabolites (nmol liter^−1^) from the first sampling period (*R *=* *0.194, *P < *0.001). (C) NMDS of the second sampling period alone: July 31st to August 3rd. The NMDS analysis results were significant (Monte Carlo randomization *P < *0.01) with a stress value of 0.17. (D) NMDS of full dataset: July 26th to August 3rd. Colors indicate time of day that the samples were collected. The NMDS analysis results were significant (Monte Carlo randomization *P < *0.01) with a stress value of 0.18. Download FIG S1, TIF file, 0.4 MB. Copyright © 2021 Boysen et al. 2021 Boysen et al. https://creativecommons.org/licenses/by/4.0/ This content is distributed under the terms of the Creative Commons Attribution 4.0 International license.

10.1128/mSystems.00896-20.8 TABLE S3 Pairwise comparisons of samples collected at different time points from the multivariate analyses of particulate metabolite concentration during the first sampling period. Download Table S3, CSV file, 0.00 MB. Copyright © 2021 Boysen et al. 2021 Boysen et al. https://creativecommons.org/licenses/by/4.0/ This content is distributed under the terms of the Creative Commons Attribution 4.0 International license.

### Metabolite diel periodicity.

**FIG 3:**
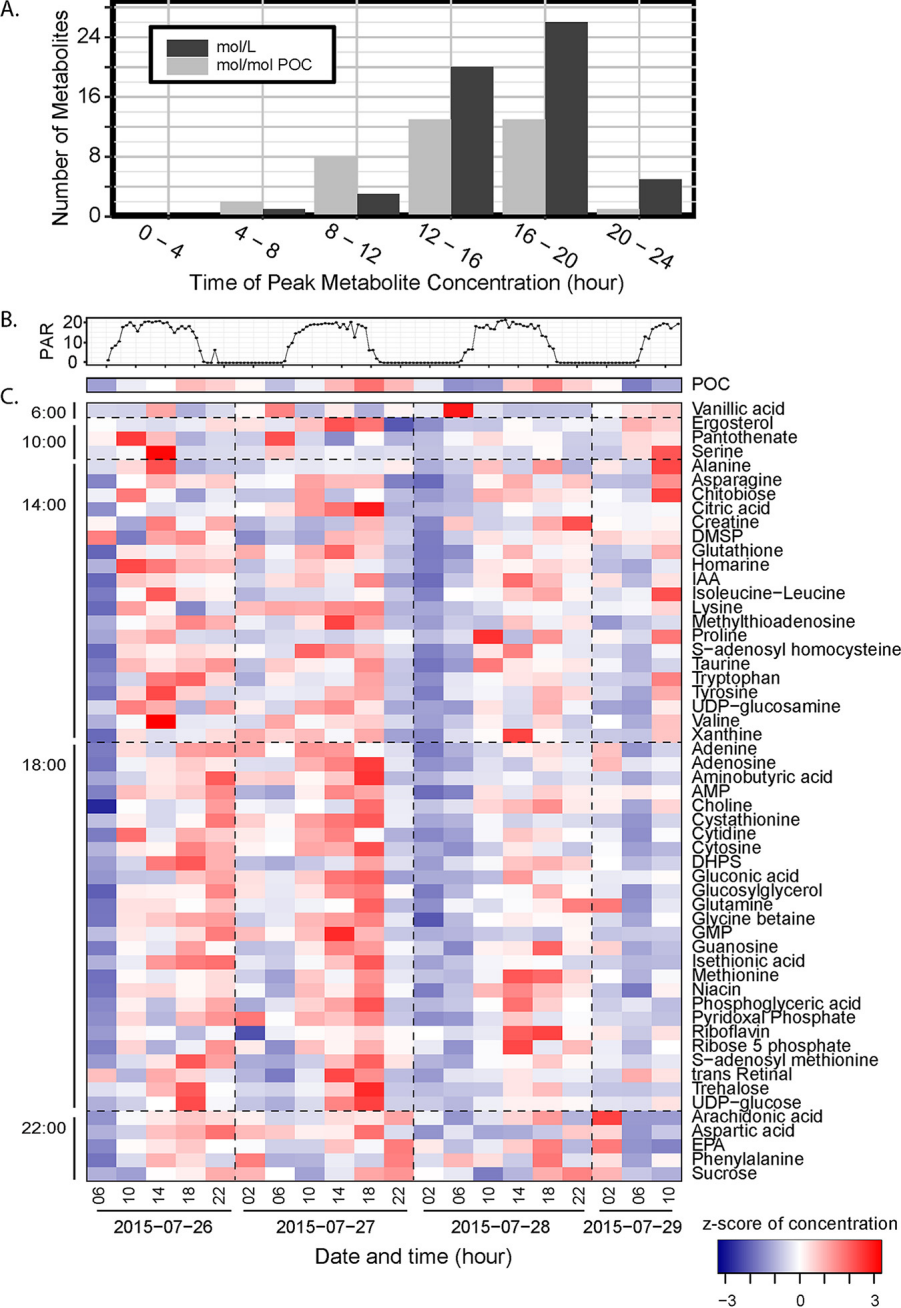
Time of day that significantly diel compounds peak in the first sampling period (A). Surface light (photosynthetically active radiation [PAR], ×10 nmol photon m^−2^ s^−1^) (B). Heat map showing the *z*-score standardized concentrations of POC and of metabolites (nmol liter^−1^) determined to be significantly diel in the first sampling period, arranged by time of peak concentration (C).

To determine whether oscillations in particulate metabolite concentrations were driven by changes in biomass or by changing cell physiology, we calculated metabolite concentrations as moles of particulate metabolite relative to water volume filtered, resulting in values of molar concentration (nmol liter^−1^), and as molar concentration relative to POC concentration, resulting in values of nmol per μmol POC. Bulk and individual metabolite concentrations oscillated both in molar concentration and relative to POC (Fig. 2; Fig. 3A and B) (Rhythmicity Analysis Incorporating Nonparametric methods [RAIN] false-discovery rate [FDR]-corrected *P < *0.05). The molar concentration (nmol liter^−1^) of 55 metabolites (70%) had significant 24-h oscillations, with 26 reaching a maximum in concentration within 2 h of 18:00 and 20 reaching their peak concentration within 2 h of 14:00 (Fig. 3A and B; Table S1). Relative to POC (nmol μmol POC^−1^), 37 compounds (47%) showed diel oscillations (Table S1), and the mean time of peak concentration shifted to earlier in the afternoon (Fig. 3A). POC reflects total community biomass and detritus, so to avoid assumptions of metabolite source, we present molar concentrations throughout except when metabolite source can be constrained to a specific phytoplankton type, in which case we present metabolite concentration relative to the cell number or biomass of the source organism.

**FIG 4:**
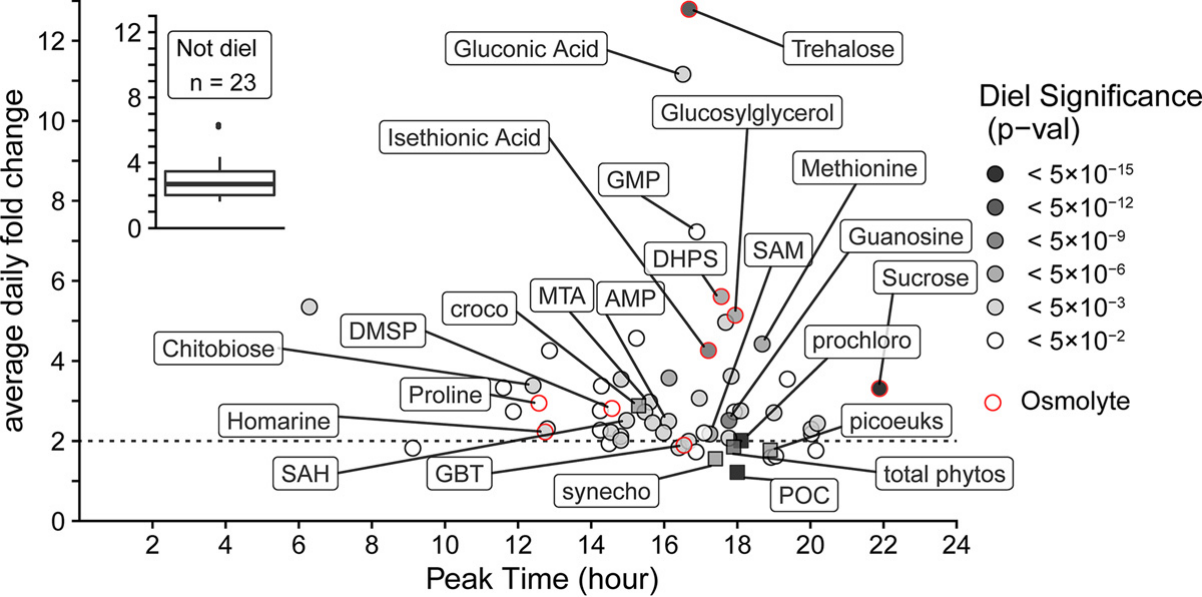
Peak time versus average daily fold change for each metabolite (circles, calculated based on concentrations proportional to nmol liter^−1^) and POC from beam attenuation and phytoplankton biomass from flow cytometry (squares, calculated based on concentrations proportional to μg C liter^−1^). Gray color indicates the level of significance (FDR-corrected *P* value) of the 24-h oscillation. Red outlines indicate that the compound is an osmolyte. Select compounds and all biomass estimates are labeled (croco, *Crocosphaera*; synecho, *Synechococcus*; prochloro, *Prochlorococcus*; picoeuks, photosynthetic picoeukaryotes; total phytos, total phytoplankton biomass from underway flow cytometry). Dashed line is at a 2-fold change, which is above that for POC and total picophytoplankton biomass. The inset shows the distribution of fold change in nonsignificant compounds. These compounds were variable in concentration over time even though they do not have significant diel oscillations.

**FIG 5:**
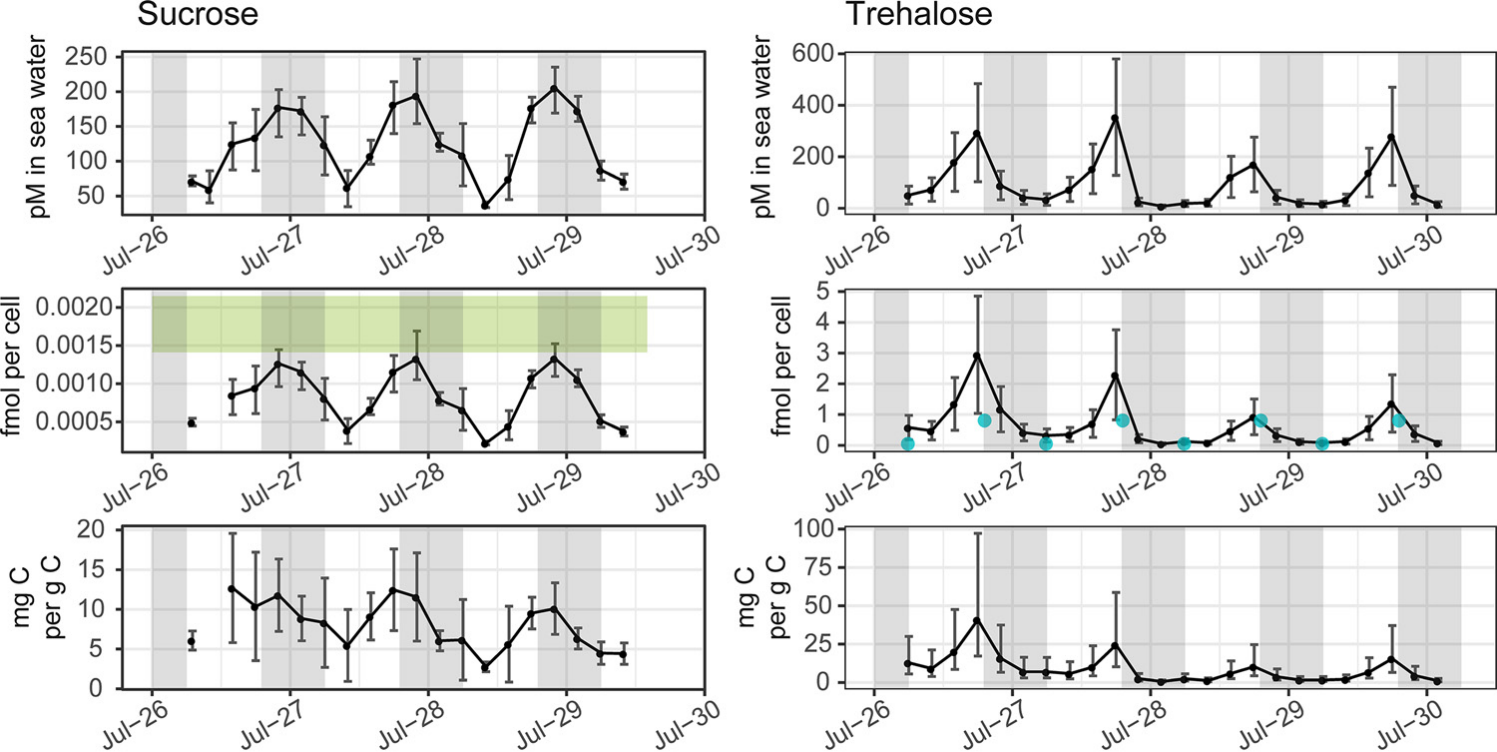
Particulate sucrose (left) and trehalose (right) measured as pmol liter^−1^ in seawater (top), fmol cell^−1^ (middle) of *Crocosphaera* and *Prochlorococcus* for trehalose and sucrose, respectively, and mg g^−1^ cell carbon (bottom) of *Crocosphaera* and *Prochlorococcus* for trehalose and sucrose, respectively. The light gray vertical shading represents nighttime. The green box in the middle-left panel indicates the range of cellular sucrose quotas measured in triplicate *Prochlorococcus* MIT1314 cultures harvested midday in exponential growth. The blue points in the middle-right panel indicate the dawn and dusk values measured for trehalose quotas in Crocosphaera watsonii WH8501. In the top panels, the error bars represent one standard deviation around the mean value, including uncertainty from the quantification regression. The error bars in the middle panels represent one standard deviation around the mean. The error bars in the bottom panels represent the 95% confidence interval given the confidence in the biomass quantification from underway flow cytometry.

**FIG 6:**
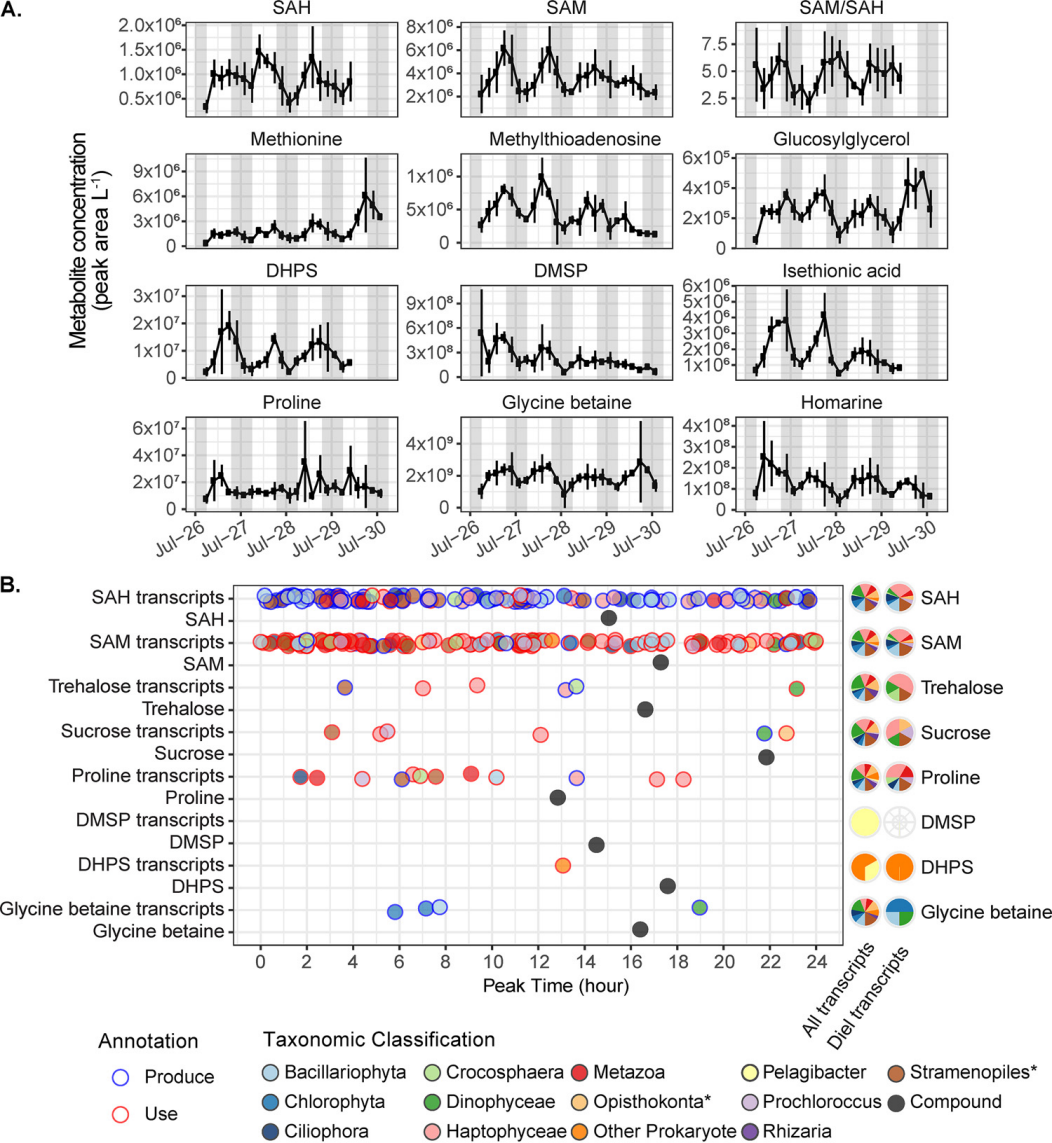
(A) Diel metabolite concentrations (peak area liter^−1^, proportional to nmol liter^−1^) of methionine-cycle compounds, methylthioadenosine, and select osmolytes. Error bars are the standard deviation of biological triplicates. The light gray vertical shading represents nighttime. (B) Left: Time of peak abundance of diel transcripts related to the production or use of select diel osmolytes and primary metabolites. Fill color indicates the phylogenetic lineage of the transcript; outline color indicates whether the transcript is associated with production or consumption of the metabolite. Time of metabolite peak concentration (nmol liter^−1^) is in black. Right: Proportion of all transcripts and diel transcripts belonging to each taxon. *, does not include select subgroups shown otherwise.

Metabolites with significant oscillations (Fig. 3C) had daily fold changes ranging from 2 to 12.8, all of which exceeded the 1.2- and 1.8-fold changes of POC and the sum of FCM-derived phytoplankton biomass, respectively (Fig. 4). These compounds function in cell turgor, anabolism, energy storage, and redox balance (5, 11, 13, 39–50). The disaccharides trehalose and sucrose displayed the most robust oscillations (*P* value < 1 × 10^−13^) (Fig. 4 and 5). Trehalose and sucrose are known osmolytes, compounds that accumulate as compatible solutes (47), and nearly all other identified osmolytes (9/10) showed diel oscillations (Fig. 4 and Fig. 6A; Table S1). Glutamic acid is the only known osmolyte that did not have a significant oscillation in molar concentration (Table S1).

Metabolites with significant diel cycles involved in cell growth include the three methionine-cycle compounds that were detected, *S*-adenosyl methionine (SAM), *S*-adenosyl homocysteine (SAH), and methionine. 5′-Methylthioadenosine (MTA) is produced from SAM during polyamine synthesis and had a temporal pattern that closely matched that of SAM (Fig. 3C and Fig. 6A), such that SAM/MTA remained relatively constant. The cofactor pantothenate (vitamin B_5_) was one of the few compounds that peaked in the morning (Fig. 3C). Cofactors riboflavin and niacin (vitamins B_2_ and B_3_) oscillated with maxima near dusk. Reduced glutathione oscillated with an afternoon peak (Fig. 3C).

### Connections between metabolites and transcripts.

To investigate the relationships between gene expression and metabolite concentration we used the Kyoto Encyclopedia of Genes and Genomes (KEGG) database to connect metabolites with transcripts annotated as encoding proteins that directly produce or degrade each metabolite. KEGG was chosen because it currently contains the greatest number of metabolites compared to that in other databases that link genes, enzymes, and metabolites (51). Using KEGG, all but four of the diel metabolites were linked to at least one annotated prokaryotic or eukaryotic transcript (Fig. S2). Glucosylglycerol, ergosterol, and isethionic acid are in the KEGG database, but no transcripts were annotated in our data set as directly producing or degrading them, while homarine is not included in the KEGG database.

10.1128/mSystems.00896-20.3 FIG S2 Diel transcript peak abundance related to the production or degradation of diel metabolites. Color indicates the phylogenetic lineage of the transcript. Left: Peak time of transcript abundance or particulate metabolite concentration (nmol liter^−1^). Right: Proportion of diel transcripts belonging to each taxa and proportion of all transcripts, regardless of diel oscillation, related to each metabolite belonging to each taxon. Download FIG S2, TIF file, 2.1 MB. Copyright © 2021 Boysen et al. 2021 Boysen et al. https://creativecommons.org/licenses/by/4.0/ This content is distributed under the terms of the Creative Commons Attribution 4.0 International license.

Transcripts provide insight into the number and identity of organisms and pathways that may be responsible for the metabolite’s synthesis and degradation, despite potential database biases and sequencing depth. The orders containing *Crocosphaera*, *Prochlorococcus*, Pelagibacter ubique, and other unclassified alphaproteobacteria comprised ∼50% of all prokaryotic transcripts that could be linked to metabolites (Data set S2). Dinoflagellates (Dinophyceae), nondiatom stramenopiles (Stramenopiles), haptophytes (Haptophyceae), nonmetazoa opisthokonts (Opisthokonta), and diatoms (Bacillariophyta) comprised ∼70% of eukaryotic transcripts linked with metabolites (Data set S2).

10.1128/mSystems.00896-20.10 DATA SET S2 Prokaryotic and eukaryotic transcripts that matched metabolites identified by organism taxa and KEGG ortholog. If the transcript is significantly diel (RAIN FDR-corrected *P* value < 0.05) the time of peak transcript abundance is provided (0/24 is midnight, 12 is noon). Download Data Set S2, XLSX file, 0.2 MB. Copyright © 2021 Boysen et al. 2021 Boysen et al. https://creativecommons.org/licenses/by/4.0/ This content is distributed under the terms of the Creative Commons Attribution 4.0 International license.

AMP, SAM, and SAH stand out as the diel metabolites with the greatest number of associated diel transcriptional patterns, with 181, 124, and 113 transcripts, respectively (Fig. 6B; Fig. S2). Most diel transcripts associated with SAM and SAH encoded methyltransferases that use SAM and produce SAH (Fig. 6; Data set S2). In most other cases, there were few diel transcripts associated with a metabolite (e.g., only 6 diel transcripts were associated with trehalose) (Fig. 6B; Fig. S2).

As a first step to investigate the temporal relationship between gene expression and metabolite concentration, we estimated the lag-time between peaks in metabolites and in transcripts that exhibited significant diel periodicity in abundance. This analysis showed a broad distribution in the lag-times between metabolites and transcripts (Fig. S3), with no significant relationship between the peak time of prokaryotic or eukaryotic transcripts and their associated metabolites (Pearson correlation *P* value > 0.05).

10.1128/mSystems.00896-20.4 FIG S3 Offset time (in hours) between the diel compounds and diel eukaryotic transcripts (top) or diel prokaryotic transcripts (bottom) that use or produce them. Diel significance of compounds was based on the first sampling period, diel significance of eukaryotic transcripts was based on the first sampling period, and diel significance of the prokaryotic transcripts was based on both sampling periods (RAIN FDR-corrected *P < *0.05). Download FIG S3, TIF file, 0.6 MB. Copyright © 2021 Boysen et al. 2021 Boysen et al. https://creativecommons.org/licenses/by/4.0/ This content is distributed under the terms of the Creative Commons Attribution 4.0 International license.

### Disaccharide osmolytes can be attributed to cyanobacteria.

We observed trehalose-related transcripts from eukaryotic phytoplankton and from *Crocosphaera* (Fig. 6). Using published *Ostreococcus* cellular trehalose concentrations (6) and picoeukaryote cell counts, we estimated that picoeukaryote contribution to trehalose was 0.2 to 3.0 pmol liter^−1^, a small fraction of environmental trehalose (274 pmol liter^−1^ at 1,800 on average; Table S2). The abundance of *Crocosphaera* (Table 1; Fig. 1) and diel oscillations in the *Crocosphaera* transcript for trehalose 6-phosphate synthase/phosphatase (Fig. 6; Data set S2) suggest *Crocosphaera* as the main contributor of trehalose during this field study. To further explore this hypothesis, we grew Crocosphaera watsonii WH8501 under a 12:12 light:dark cycle and measured 0.8 and 0.07 fmol trehalose cell^−1^ at the end of the light and dark periods, respectively (Fig. 5). Given the *Crocosphaera* abundance during our sampling and assuming similar intracellular concentration, this accounts for 1.8 to 670 pmol liter^−1^ particulate trehalose, comparable to total particulate trehalose during our sampling (2.8 to 627 pmol liter^−1^ across both sampling periods; Fig. 5).

Multiple taxa expressed transcripts related to production and degradation of sucrose, including *Prochlorococcus* (Fig. 6). To assess the potential contribution of *Prochlorococcus* to environmental sucrose concentrations, we measured the cellular sucrose quota in a culture of *Prochlorococcus* MIT1314 harvested midday during exponential growth. Using the cellular quota of sucrose in these cultures (range in biological triplicates: 1.4 to 2.1 amol cell^−1^) and the abundance of *Prochlorococcus* during the environmental sampling, it is possible that all the observed sucrose could have been in *Prochlorococcus* during this study (Fig. 5).

## DISCUSSION

As a whole, the metabolites we measured comprise up to 2% of POC and 3% of PN in our samples (Fig. 2B and C). This contribution fits within the bounds of a POC budget, given that ∼80% of surface POC is comprised of lipid, carbohydrate, and protein macromolecules (1, 2) and that DNA, RNA, and pigments each contribute several percentages of the dry weight of actively growing microalgae (52). Metabolite pools are dynamic, and an increase in the concentration of a given metabolite over time suggests that sources of that compound (anabolism, regeneration, uptake from dissolved pools, or polymer disassembly) are greater than sinks (catabolism, use, exudation, cell death, or polymer assembly). Assuming static enzyme concentrations and activity, higher substrate concentrations result in higher enzyme reaction rates. Thus, elevated metabolite pool size favors the maintenance of high cellular activity. In our study, the highest metabolite concentrations corresponded with the daily peak in biomass near the end of the light period. Diverse organisms (including heterotrophs and autotrophs) expressed diel cycles in the abundance of those transcripts related to the production and degradation of diel metabolites, but the timing of peaks in diel transcript abundances was not a reliable indicator of metabolite levels, consistent with previous work showing that diel protein abundances and transcripts were not closely linked (25). There are diverse processes and opportunities for cellular regulation that could occur between transcription and metabolite production, with posttranslation modification of enzymes as a single example. Nevertheless, the pairing of diel measurements of metabolites and transcripts allows investigation of how many and which organisms and processes may be responsible for the accumulation and depletion of a given compound.

### Diel partitioning of anabolism, catabolism, and redox maintenance.

The diel oscillations in POC and FCM-resolvable phytoplankton biomass reflect the alternation of carbon fixation, anabolism, and growth during daylight hours and respiration, catabolism, and mortality during the night (Fig. 1) (17, 19, 27). The community metabolome reflected these patterns, with an overall increase in concentration throughout the day while cells are growing (Fig. 2). Macromolecular measurements show that phytoplankton use sugars and lipids overnight (22, 26, 28, 32, 46) and synthesize protein in the early morning in order to optimize photosynthesis during the day (31). The observations of diel oscillations in primary metabolites here highlight the timing and extent of this preparation in a natural environmental community.

Multivariate similarity of samples collected at 6:00 a.m. indicates a consistent morning phenotype with low concentrations of metabolites (see Fig. S1A and B in the supplemental material), reflecting nighttime use of energy stores and recovery from daytime oxidative stress (46). Nearly half of the diel metabolites (26/55) had peak molar concentrations near dusk (Fig. 3), corresponding with a peak in carbon biomass. However, for most (46/55) diel metabolites, the daily enrichment of a metabolite exceeded that of POC or total FCM-resolvable phytoplankton biomass, which had daily fold changes of 1.2 and 1.8, respectively (Fig. 4). This suggests that these metabolites likely had oscillations in intracellular concentration, as previously observed for many primary metabolites in non-marine cyanobacteria (45).

Primary metabolites are particularly powerful indicators of biochemical activity on the community scale. SAM, SAH, and AMP are compounds involved in biosynthesis and growth that had diel oscillations with daytime increases (Fig. 3 and 6). Transcripts associated with these molecules displayed diel oscillations in abundance, with peaks at different times of day, across myriad pathways and microbial taxa (Fig. 6; Fig. S2). Despite this diversity in use, the sum of community activity was reflected in diel oscillations of metabolite concentrations, which were synchronized with daytime biomass accumulation. Further evidence of this daytime community-scale anabolism is the diel oscillation of pantothenate (vitamin B_5_), a component of coenzyme A as well as acyl carrier protein. Pantothenate peaked in the morning (Fig. 3), suggesting that the community was poised to assemble these cofactors for daytime biosynthesis.

SAM is a ubiquitous methyl donor used by all living cells. During methylation, SAM is converted to SAH, which is then regenerated back to SAM via methionine. In addition to its role in methylation, SAM is essential for polyamine synthesis and is the most common riboswitch effector in prokaryotes (53). SAM riboswitches have been observed in native Station ALOHA bacterioplankton populations (54). SAH had an afternoon peak time, such that the SAM/SAH ratio was at a minimum during the day (Fig. 6). This ratio reflects methylation potential (55), suggesting that the demand for methylation outstripped the supply of SAM in the light despite rising intracellular concentrations of SAM during the day, concentrations which likely aid in maintaining high reaction rates. Over the dark period, SAM/SAH ratios recovered, suggesting that catabolic processes dominated and the need for SAM was diminished. Many cells require cobalamin (vitamin B_12_) to catalyze the reactions that regenerate methionine, and SAH is elevated relative to SAM during cobalamin stress as cells struggle to complete the cycle (11). Thus, it is possible that the lower SAM/SAH ratio additionally reflects a daytime increase in cobalamin demand among auxotrophic community members.

All living cells produce reactive oxygen species, and redox homeostasis is a critical part of maintaining cellular function. Reactive oxygen species produced during photosynthesis accumulate over the day and present a continuing challenge for cells at night (40). Strategies for managing oxidative stress range from the acute reactions that detoxify reactive oxygen species to mitigation and avoidance strategies, such as accumulating glycogen to assimilate excess reducing power in high-light conditions and avoid dangerous levels of membrane redox potential (46). Reduced glutathione is the active form of a key component of the reactive oxygen species detoxification system and peaked during the afternoon (Fig. 3B), as has been observed in cultures and field studies (41). The daytime peak possibly reflects production to compensate for increased oxidative stress during the day and a subsequent decrease in production and oxidation of the residual pool overnight.

Riboflavin and niacin (vitamins B_2_ and B_3_) are precursors to cofactors flavin mononucleotide/flavin adenine dinucleotide (FMN/FAD) and NAD/NADP, respectively. These cofactors are involved in electron transport chains for photosynthesis and respiration and are therefore key components of redox processes within cells. The daytime accumulation of riboflavin and niacin (Fig. 3; Table S1) underscore that the community-wide processes of growth metabolism and redox maintenance occur in step with the diel cycle.

### Diel oscillations in osmolyte concentrations reveal their functional diversity.

Metabolites with osmolyte properties are among the most abundant compounds within marine microbial cells (5, 13, 44, 47, 48, 56) and exhibited diel oscillations in concentration (Fig. 5 and 6). One exception to this observation was glutamic acid, which plays other critical roles as a component of protein and in regulating nitrogen assimilation in addition to its osmotic properties (47). In the absence of fluctuations in salinity or temperature, oscillations in osmolyte concentrations occurred in excess of or out of sync with biomass oscillations and point to alternative roles for this compound group such as previously hypothesized roles in short-term energy and nutrient storage (47) (Fig. 4; Table S1). Intracellular accumulation of metabolites occurred predominantly during the day when electron flow through the photosystems and the Calvin cycle exceeds that required to maintain maximum division rates. The resulting need to dissipate reductant is typically channeled into the production of carbohydrates like glycogen (22, 39, 46), into exopolymeric substances (57, 58), or into storage lipids (26, 32). Cyanobacteria, for example, manage excess energy during the day by both storing glycogen and producing small molecules that can either be stored or excreted (39, 40, 46, 49, 50). These energy stores are used to fuel cellular respiration and other activities at night, such as protein synthesis and preparing cells for photosynthesis (26, 31, 32, 39, 46). Unlike starch and storage lipids, osmolytes do not necessarily need to go through hydrolysis, β-oxidation, or glycolysis prior to entering the tricarboxylic acid (TCA) cycle and could be used as readily available substrates for energy production and as biosynthetic intermediates while macromolecular pools are being mobilized by the cell (49).

Trehalose was the most prominent diurnally oscillating compound with diel oscillations in concentration in seawater and relative to POC (Fig. 4 and 5; Table S1). Trehalose is an osmolyte produced by the unicellular diazotroph *Crocosphaera* (42, 44), some heterotrophic bacteria, and some phytoplanktonic picoeukaryotes, including *Ostreococcus* (6). Transcriptomic evidence motivated us to measure trehalose in cultures of *Crocosphaera*, which was more concentrated at the end of the day than at the beginning of the day, similar to what we saw in the environment. Assuming trehalose in the environment is produced primarily by *Crocosphaera*, our results strongly suggest that intracellular trehalose concentrations have diel oscillations in the field (Fig. 5).

*Crocosphaera* temporally separate photosynthesis and nitrogen fixation to protect nitrogenase from oxygen (59–61), as reflected in their gene expression for photosynthesis during the night and early morning and nitrogen fixation at dusk (21). To draw down cellular oxygen and fuel nitrogen fixation, *Crocosphaera* need a nighttime energy source (62, 63). *Crocosphaera* has at least one gene encoding a protein homologous to glycoside hydrolases, family 15 (64), which contains enzymes that hydrolyze a variety of glycosidic bonds, including trehalose. Thus, it is possible that *Crocosphaera* uses trehalose as a fuel for generating the electrons and ATP required for nitrogen fixation. Using the stoichiometry of these reactions (62, 65), we estimated that trehalose catabolism could have fueled 9 to 28% of the nighttime nitrogen fixation during this expedition (calculation in supplemental material calculation on trehalose fueling nitrogen fixation) (21). As much as 60% of total dark respiration by *Crocosphaera* is used to draw down cellular oxygen rather than to directly fuel nitrogen fixation (62), and, if we adjust our calculation accordingly, trehalose can produce 3.6 to 11% of the required respiratory substrates needed for *Crocosphaera* to effectively fix nitrogen at the rates measured (21). In addition to providing energy, trehalose could be providing the carbon needed to generate TCA cycle intermediates when biosynthesis of other molecules, such as amino acids, is consuming those intermediates.

The flux of carbon through trehalose may be an indicator of the accumulation and degradation of a larger glycogen pool that accumulates during the day and is used at night (66). Shi et al. (67) suggest that *Crocosphaera* cells are depleted of storage compounds at night, since prolonged dark does not result in increased nitrogen fixation. If this hypothesis is correct, the total amount of nitrogen fixation possible is limited by the amount of energy stored in substrates such as trehalose and glycogen during daytime, and the ability to accumulate and use these compounds could have impacts on the nitrogen budget of the microbial community.

Another disaccharide osmolyte, sucrose, displayed an oscillation with a maximum daily concentration at 22:00 when calculated relative to seawater and to POC (Table S1). Sucrose is the major compatible solute in high-light *Prochlorococcus* (56), and the observed environmental variation may reflect the *in situ* accumulation and use of glycogen by *Prochlorococcus*. Though other organisms also expressed sucrose-related genes (Fig. 6), *Prochlorococcus* was the numerically dominant sucrose-producing organism detected in these populations (Table 1). *Prochlorococcus* has strong diel gene expression for anabolic and catabolic processes (23) and is known to accumulate polysaccharides during the day, particularly under nitrogen limitation (68). If we assume that cellular quotas of sucrose in *Prochlorococcus* grown in culture are similar to those in the environment, *Prochlorococcus* alone could explain the sucrose concentrations seen in the environment (Fig. 5). Sucrose had a diel oscillation when calculated relative to *Prochlorococcus* cell counts and biomass (Fig. 5). These potential intracellular oscillations lead us to hypothesize that *Prochlorococcus* uses sucrose for energy storage and not only as a compatible solute, as has been observed in nonmarine cyanobacteria (45, 49).

Homarine and DMSP are known eukaryotic osmolytes (5, 47, 48, 69). The amplitude and timing of the diel oscillations in these two compounds differ from those observed in phytoplankton picoeukaryote biomass (Fig. 4), again suggesting that these compatible solutes play multiple roles within the microbes that use them as osmolytes. This diversity of functions is well established for DMSP, which influences grazing behaviors and can function as an antioxidant (5, 43). DMSP is also a source of carbon and reduced sulfur in the microbial community, with uptake and assimilation both tied to light availability (70, 71). In our analysis, the only annotated transcript related to DMSP encodes a SAR11 DMSP demethylase required for DMSP degradation (72) (Fig. 6). A dearth of data on the roles of homarine in marine microbes and a lack of genetic information about homarine synthesis and degradation limit our ability to infer the sources and sinks for this abundant compound. The high concentration and diel dynamics of homarine call for further investigation.

Both isethionic acid and DHPS are associated with fast-growing eukaryotes that need to mobilize cellular machinery to transport materials into the mitochondria for respiration (13, 73), and recent work has suggested that DHPS has potential osmotic capabilities (13). These two metabolites had large diel oscillations, implicating them as temporary stores of energy or intermediates that can be mobilized quickly. Our data implicate SAR11 and *Rhodobacteraceae* as likely DHPS degraders at Station ALOHA (Fig. 6), although genes for the production of DHPS are not in the KEGG database and thus were not identified by our analyses. If production and degradation of these compounds are separated along phylogenetic lines (36), then these compounds are likely excreted into the dissolved phase by eukaryotes and subsequently available for use by bacteria, as suggested in Durham et al. (13). This may explain the midday maximal expression of an *hpsN*-like *Rhodobacteraceae* DHPS degradation gene (Fig. 6).

Glycine betaine is an osmolyte found within many marine microorganisms and can play multiple cellular roles, including modulating buoyancy, acting as a methyl donor, and providing a nitrogen source (5, 47, 74). Glycine betaine had a significant diel oscillation with an evening peak and similar fold change to that of the total FCM-quantifiable biomass (Fig. 4). *Chlorophyta*, *Dinophyceae*, and diatoms expressed glycine betaine synthesis genes with diel periodicity while many more groups expressed related genes that lacked diel periodicity (Fig. 6). It is possible that osmotic balance is maintained in certain phytoplankton by the relatively stable intracellular concentration of glycine betaine while other osmolytes are more dynamic pools with higher daily fold change.

### Metabolites as fuel for the microbial loop.

Although our data suggest that over diel cycles many metabolites are respired directly by the phytoplankton that produce them, other metabolites quantified here are known to fuel heterotrophic bacterial growth in marine ecosystems (71, 75–78). DMSP, for example, can support up to 9.5% of the bacterial carbon demand at Station ALOHA (70). Additionally, glycine betaine can support heterotrophic bacterial growth, and both natural marine populations and isolated bacteria are known to have high-affinity glycine betaine transporters (79–81). The oscillations of particulate metabolite concentrations observed in this study call for further investigation into the hypothesis that these compounds are important substrates for community interactions and resources for the microbial loop. For compounds that exhibited diel oscillations, the difference between the daily maximum and minimum values provides a daily net production and degradation rate. We estimated a total net turnover rate of over 32 nmol C liter^−1^ d^−1^ from our targeted metabolites, with several metabolites exhibiting individual turnover rates of over 1 nmol C liter^−1^ d^−1^, including arachidonic acid, trehalose, homarine, sucrose, glycine betaine, glucosylglycerol, and DHPS (Table S1). These are conservative estimates since the instantaneous flux may be much higher than the daily net change and we did not measure excretion of metabolites into the dissolved pool. For example, dissolved DMSP has a turnover time of 4.5 h at Station ALOHA (70) and has been shown to be produced at night and during the day (82). Both of these observations about DMSP would substantially increase the baseline estimate of DMSP production made here, which does not account for rapid turnover and only includes a daytime increase in intracellular concentration.

While the fate of the metabolites measured here remains unclear, conservative estimates of carbon and nitrogen flux through these small pools was large, comprising around 2% of the ^14^C based estimates of primary productivity during this study (26). These compounds are potentially used for cellular requirements by the organisms synthesizing them, as discussed above, or released into the labile dissolved pool. When they enter the dissolved pool through excretion or cell lysis, these compounds are important components of the labile dissolved organic matter pool (77) and play a role in organism interactions (83, 84).

### Conclusions.

The light-dark cycle plays a dominant role in structuring marine microbial activity. Previous work has shown diel oscillations of community processes, such as daily accumulation and depletion of POC (27), and diel oscillations of transcriptional activity, which have provided new information on temporal dynamics and raise hypotheses about the activity of individual taxa (23, 24). Measurements of *in situ* metabolites in native planktonic microbial populations reported here support the hypotheses that diverse microbial taxa in the NPSG are synchronized to daily oscillations of light energy and photosynthesis, with metabolites accumulated during the day and depleted at night. The diel oscillations of ubiquitously used primary metabolites are a direct manifestation of photoautotrophic organisms dominating the community and driving anabolic processes during the day and catabolic processes at night. The combination of transcript abundances, metabolite concentrations, and taxa-specific biomass in the field and in culture allows us to postulate that *Crocosphaera* uses trehalose as a short-term energy source to drive nighttime nitrogen fixation. Trehalose and the other osmolytes we measured are highly abundant in cells and, in addition to playing multiple roles within producers, likely fuel respiration in heterotrophic bacteria. Studies in model organisms suggest mechanisms for why some common metabolites, such as niacin and riboflavin, have diel oscillations, but the hypotheses presented here need to be validated with future studies. Though metabolic flux is often regulated at other points along metabolic pathways rather than at the individual transcript levels, measuring gene expression remains one of the most useful ways we have of probing a community’s metabolic state. However, metabolite concentrations cannot be predicted from transcripts in a single organism in pure culture, let alone in a complex natural community. Pairing quantitative measurements of particulate metabolites with transcriptomes is a key step toward understanding how regularly oscillating gene expression in microbial communities is reflected in the net community processes we observe and further elucidates the currencies of the microbial community.

## MATERIALS AND METHODS

### Sample collection.

Samples were collected on the R/V *Kilo Moana* in the NPSG (near 24.5° N, 156.5° W) every 4 h for two sampling periods in summer 2015 (period one: July 26, 6:00 to July 30, 6:00; period two: July 31, 18:00 to August 3, 18:00). To limit variability unrelated to solar forcing, we conducted Lagrangian sampling following two drifters in an anticyclonic eddy (21). Samples for particulate metabolites and transcripts were collected from 15 m water depth using Niskin bottles attached to a conductivity, temperature, depth array (CTD). Ancillary measurements for nutrients and heterotrophic bacterial abundance (reported in Wilson et al. [21]) were collected and analyzed with standard Hawaii Ocean Time-series protocols (http://hahana.soest.hawaii.edu/index.html).

### Bulk and taxa-specific carbon biomass.

POC concentrations were derived from particulate beam attenuation at 660 nm measured via a hyperspectral absorbance and attenuation meter (ac-s, Wetlabs, as published in White et al. [27]). Particle attenuation at 660 nm (c_p_ 660, m^−1^) was calibrated against discrete POC samples taken near dawn and dusk (*n *=* *30; *r* of a type II regression = 0.78). Discrete POC and PN samples were collected by filtration of the ship’s underway flow through seawater onto combusted GF/F filters. Analysis is further described in the section on particulate carbon and particulate nitrogen in Text S1.

10.1128/mSystems.00896-20.1 TEXT S1 The supplemental text includes additional details of metabolite extraction, data collection, and data processing. There are additional details of particulate carbon and nitrogen sample collection and processing, details of multivariate analyses, and details of phytoplankton culture conditions. This text includes the supplemental calculation of how much nitrogen fixation by *Crocosphaera* could be fueled by trehalose catabolism. Supplemental discussions related to the multivariate analyses results and regarding the decrease in the number of metabolites that had significant diel cycles during the second sampling period are also included. Download Text S1, DOCX file, 0.04 MB. Copyright © 2021 Boysen et al. 2021 Boysen et al. https://creativecommons.org/licenses/by/4.0/ This content is distributed under the terms of the Creative Commons Attribution 4.0 International license.

Continuous underway flow cytometry (SeaFlow) (85) was used to count *Prochlorococcus*, *Synechococcus*, picoeukaryotes (eukaryotic phytoplankton 2 to 4 μm in size), and *Crocosphaera*. These data were supplemented with discrete flow cytometry sample analysis as in Wilson et al. (21). Cell diameters of individual cells were estimated from light scatter by the application of Mie theory (86) to a simplified optical model and converted to carbon quotas assuming spherical particles, as described in Ribalet et al. (87). Carbon biomass was estimated by multiplying cell abundance by carbon quotas.

### Metabolite extraction, data acquisition, and processing.

Metabolite samples were collected in triplicate at each time point by filtering 3.5 liters of seawater onto a 47 mm by 0.2 μm polytetrafluoroethylene (PTFE) filter (Omnipore) using a peristaltic pump, polycarbonate filter holder, and Masterflex PharMed BPT tubing (Cole-Parmer). Filters were frozen in liquid nitrogen immediately after filtration and stored at −80°C. Metabolite extractions employed a modified Bligh-Dyer method (4, 13, 88), resulting in aqueous and organic soluble metabolites with heavy stable isotope-labeled extraction and injection internal standards added to both fractions (Text S1, “metabolite sample extraction”). Unused filters extracted alongside the samples served as methodological extraction blanks.

Metabolomics data were collected by paired liquid chromatography mass spectrometry (LC-MS) with a Waters Acquity I-Class ultraperformance liquid chromatography (UPLC) system and a Waters Xevo TQ-S triple quadrupole with electrospray ionization in selected reaction monitoring mode with polarity switching, targeting over 200 compounds (4). Two separate analysis were performed for each sample, one using hydrophilic liquid interaction chromatography and the other using reversed phase chromatography. The software Skyline was used to integrate LC-MS peaks (89), and resulting peak areas were normalized to the peak area of internal standards using best-matched internal standard normalization to reduce variability introduced through the extraction and analysis process (4). Detection limits for the metabolites using these methods are published in Boysen et al. (4). A subset of these data are presented in Durham et al. and Muratore et al. (13, 29).

Metabolites with isotopologue internal standards were quantified in all samples. Trehalose, sucrose, and 2,3-dihydroxypropane-1-sulfonate (DHPS) were quantified with standard additions. For all other metabolites (Table S1), concentration (pmol liter^−1^) was calculated from injections of known concentrations of authentic standards in both water and a pooled sample from filtered euphotic zone seawater from the same cruise to correct for ion suppression. Dimethylsulfoniopropionate (DMSP) loss is known to occur during methanol-based extractions, so concentrations are considered a minimum estimate (90). Details are in Text S1 in the section on the quantification of select metabolites. The amount of each metabolite in each sample is presented in units of metabolite concentration where the amount is normalized to liters of water filtered (nmol metabolite/liter) and percent POC or percent PN where the amount is normalized to POC or PN (percent POC and percent PN calculated as mol C or N in metabolite/mol C or N in POC or PN ×100).

### Metatranscriptome data acquisition and processing.

Whole community transcript data are referred to here as prokaryotic transcript data, as they were enriched in bacterial and archaeal RNA. These metatranscriptome samples were collected on 0.2-μm filters simultaneously with the metabolomic data reported here, and the sample collection and processing have been described in Wilson et al. (21) and Aylward et al. (20). Briefly, the metatranscriptome sequence reads were quality trimmed, end-joined, mapped, and quantified with molecular standards. Metatranscriptome sequence reads were aligned to the ALOHA gene catalog (91) using LAST version 959 (92). Sequence reads were quantified using transcript count normalization, leveraging molecular standards as described in Gifford et al. (93). Sequence reads were summed if assigned to the same taxonomic order and Kyoto Encyclopedia of Genes and Genomes (KEGG) orthologue (94).

Poly A+ selected transcript data (referred to here as eukaryotic transcript data) are from the metatranscriptomes presented in Durham et al. (13). These samples were collected on 0.2-μm filters concurrently with the metabolomic samples and include only the first sampling period. Quality-controlled short reads were assembled using Trinity *de novo* transcriptome assembler version 2.3.2 (95) on the Pittsburgh Supercomputing Center’s Bridges Large Memory system. Parameters include using *in-silico* normalization, a minimum k-mer coverage of 2, and a minimum contig length of 300. The raw assemblies were quality controlled with Transrate version 1.0.3 (96). To eliminate redundancy and duplication, the assemblies were merged and clustered at the 99% amino acid identity threshold level with linclust in the MMseqs2 package (97). Using DIAMOND version 0.9.18 (98), translated eukaryotic contigs were aligned to a reference sequence database of marine organisms that includes peptide sequences from hundreds of marine eukaryotic transcriptomes (MarineRefII reference database, http://roseobase.org/data/, with additions discussed in Text S1 in “eukaryotic metatranscriptome reference database”). Taxonomy was assigned with DIAMOND by using the top 10% of hits with E value scores below 10^−5^ to estimate the lowest common ancestor of each contig. We assigned putative function using hmmsearch (from HMMER 3.1b2 [99], minimum bitscore 30) to find the best-scoring KEGG gene family from KOfam and linked the specific KO term associated with the KOfam to the contig (version 2019-03-20) (100). Contig abundances were quantified by pseudoalignment of the paired reads to the assemblies with kallisto (101) and normalized to the total number of assigned reads for a taxonomic group. Sequence reads assigned to the same taxonomic group and KEGG ortholog were summed.

Metabolites and transcripts were associated with one another using the KEGG database as a scaffold to match metabolites with transcripts coding for enzymes that directly use or produce those metabolites. The R package KEGGREST (102) was used to access the KEGG database followed by manual curation of these matches.

### Detecting periodicity.

Diel periodicity was evaluated for all signals using Rhythmicity Analysis Incorporating Nonparametric methods (RAIN) (20, 21, 103). Metabolites and transcripts were considered significantly periodic if they had a false-discovery rate (FDR) (104) corrected *P* value of <0.05. For each significantly oscillating signal, the time of peak abundance was estimated by fitting a periodic function (Text S1, “detecting periodicity and estimating time of peak concentration”), recognizing that the precision of these peak times is limited by sampling resolution. Diel periodicity in metabolites was identified for the two different sampling periods independently and jointly.

### Phytoplankton culture conditions.

Cultures of phytoplankton were grown in combusted borosilicate tubes in diurnal incubators with a 12:12 light:dark cycle. Crocosphaera watsonii strain WH8501 was grown at 27°C with 50 μmol photons m^−2^ s^−1^ in YBC-II artificial seawater medium (105) supplemented with 0.9 mM nitrate; exponentially growing cells were collected just before the lights turned on and just after the lights turned off. Cells were enumerated via a Beckman Z2 Coulter counter. *Prochlorococcus* MIT1314 (HLII clade [106]) were grown at 20°C with 20 μmol photons m^−2^ s^−1^ in Pro99 media (107) prepared with Turk Island salt solution and supplemented with 6 mM sterile sodium bicarbonate and 1 mM N-Tris(hydroxymethyl)methyl-3-aminopropanesulfonic acid (108). *Prochlorococcus* cells were collected 6 h into the light period during exponential phase and enumerated using the flow cytometer BD Influx cell sorter. Axenicity of *Prochlorococcus* cultures was verified regularly with SYBR-staining and flow cytometry (FCM) and plating on bacterial 1/2 yeast tryptone sea salts (YTSS) agar. Samples for metabolomics were collected by gentle filtration onto 0.2-μm hydrophilic polyvinylidene fluoride (Durapore) filters using combusted borosilicate filter towers.

### Data availability.

Information for the KM1513/HOE Legacy II cruise can be found online at http://hahana.soest.hawaii.edu/hoelegacy/hoelegacy.html. Raw sequence data for the diel eukaryotic metatranscriptomes are available in the NCBI Sequence Read Archive under BioProject ID PRJNA492142. Raw sequence data for the prokaryotic metatranscriptomes are available in the NCBI Sequence Read Archive under BioProject ID PRJNA358725. Raw and processed metabolomics data are available in Metabolomics Workbench under Project ID PR000926.

10.1128/mSystems.00896-20.5 FIG S4 Time of day that compounds peak in the second sampling period. Very few metabolites had significant oscillations in their concentration (nmol liter^−1^) in the second sampling period, but many metabolites had significant diel oscillations with morning peaks when calculated relative to POC because POC itself still had diel periodicity with an evening peak and morning minimum (Fig. 1). Download FIG S4, TIF file, 0.06 MB. Copyright © 2021 Boysen et al. 2021 Boysen et al. https://creativecommons.org/licenses/by/4.0/ This content is distributed under the terms of the Creative Commons Attribution 4.0 International license.
