# Correction for Boysen et al., “Particulate Metabolites and Transcripts Reflect Diel Oscillations of Microbial Activity in the Surface Ocean”

**DOI:** 10.1128/msystems.01083-22

**Published:** 2023-03-21

**Authors:** Angela K. Boysen, Laura T. Carlson, Bryndan P. Durham, Ryan D. Groussman, Frank O. Aylward, François Ribalet, Katherine R. Heal, Angelicque E. White, Edward F. DeLong, E. Virginia Armbrust, Anitra E. Ingalls

## AUTHOR CORRECTION

Volume 6, no. 3, e00896-20, 2021, https://doi.org/10.1128/mSystems.00896-20. After publication, we discovered that the authentic standard of homarine that we purchased from a commercial supplier (Santa Cruz Biotechnology, lot B1916) was contaminated with picolinic acid. This contamination resulted in an overstatement of the actual homarine concentration in these samples. The correct values for the homarine concentration are 1/25 of the previous values, based on both a commercially purchased isotope labeled standard and a newly purchased homarine standard. These values are reflected in the updated Fig. 2, Table S1, and Table S2. This change does not alter our interpretations of the data or any of the conclusions from this study.

**Figure 1:**
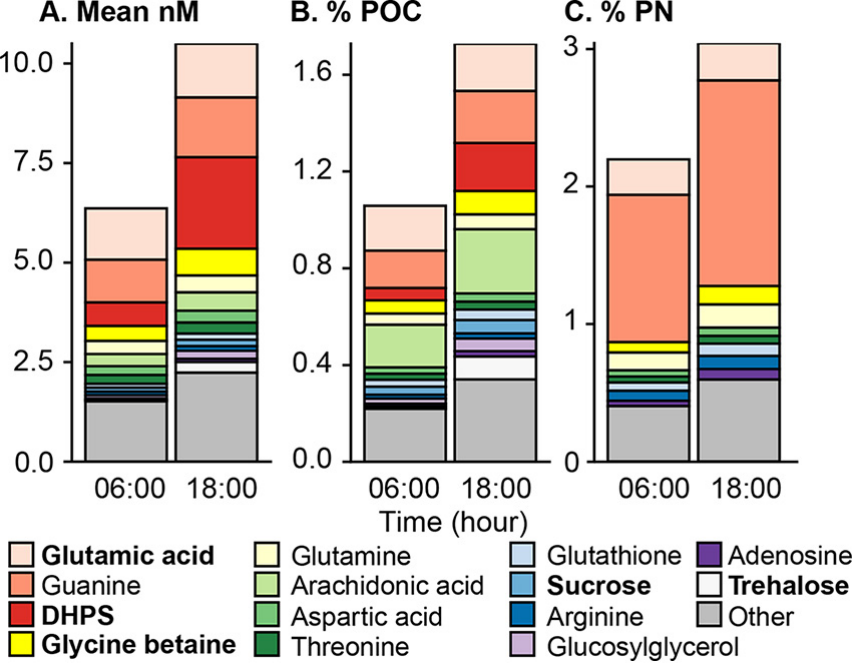


10.1128/msystems.01083-22.1 TABLE S1 Metabolites measured in this analysis. Download Table S1, CSV file, 0.01 MB. Copyright © 2023 Boysen et al. 2023 Boysen et al. https://creativecommons.org/licenses/by/4.0/ This content is distributed under the terms of the Creative Commons Attribution 4.0 International license.

10.1128/msystems.01083-22.2 TABLE S2 Average and standard deviation of targeted metabolite composition. Download Table S2, CSV file, 0.01 MB. Copyright © 2023 Boysen et al. 2023 Boysen et al. https://creativecommons.org/licenses/by/4.0/ This content is distributed under the terms of the Creative Commons Attribution 4.0 International license.

Page 4: Fig. 2 should appear as shown below.

Supplemental material: Tables S1 and S2 should appear as in the versions posted with this correction.

