# Supplementary figures and images for: Particulate Metabolites and Transcripts Reflect Diel Oscillations of Microbial Activity in the Surface Ocean
Source: mSystems. 2021 May 4;6(3):e00896-20. doi: 10.1128/mSystems.00896-20 (PMC8269247; doi:10.1128/mSystems.00896-20)

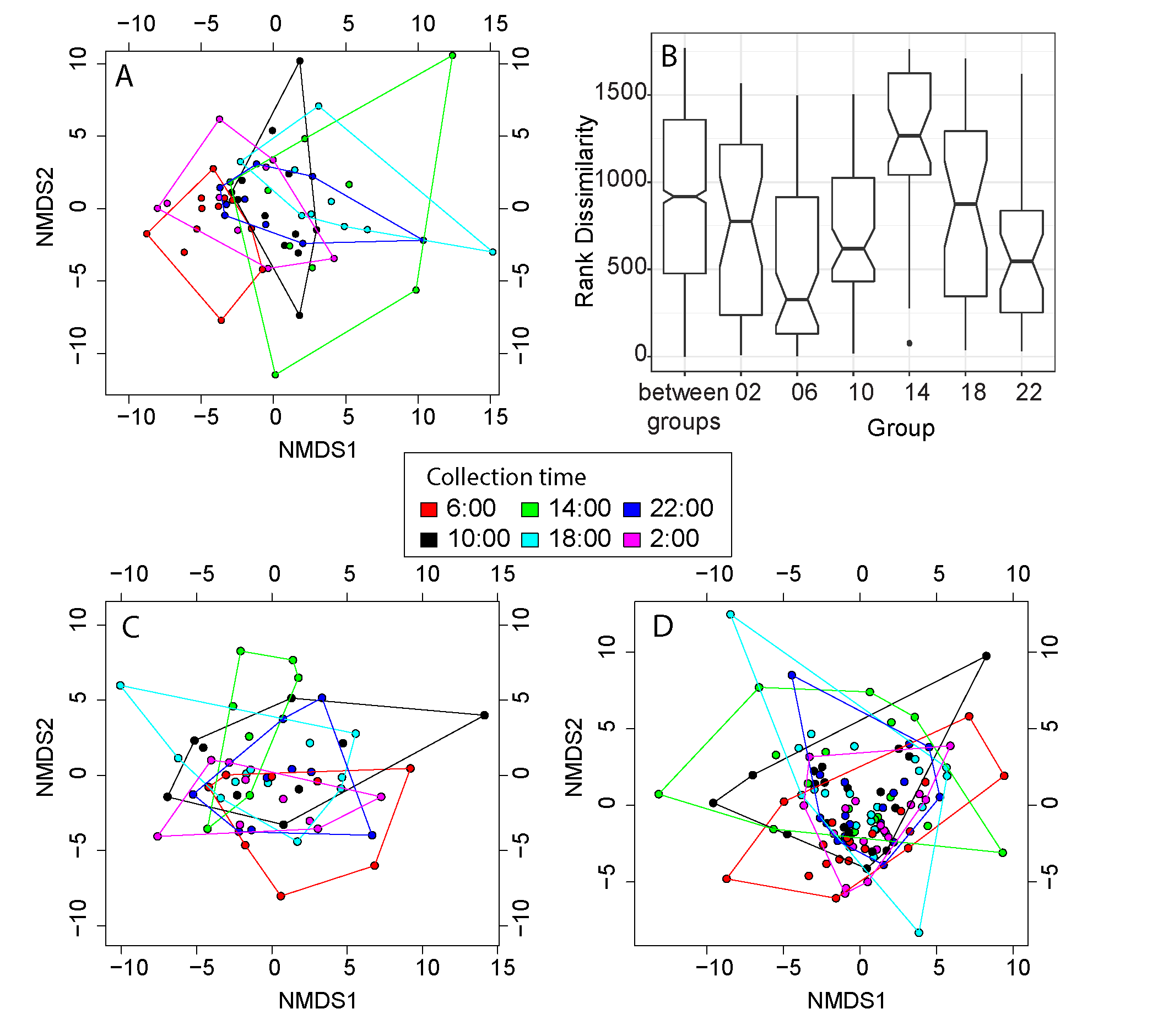

Supplement: FIG S1 [file msystems.00896-20-sf001.tif]

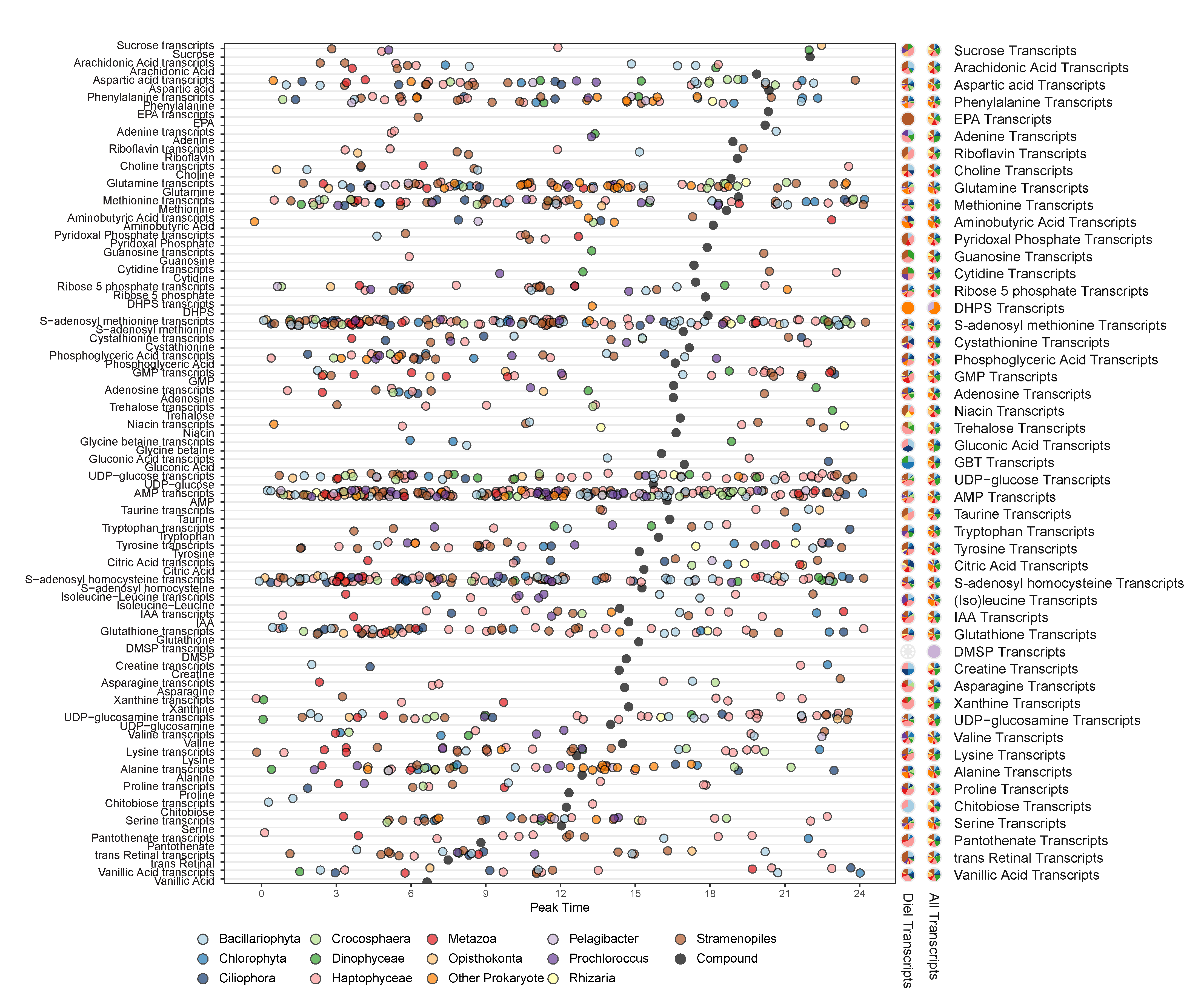

Supplement: FIG S2 [file msystems.00896-20-sf002.tif]

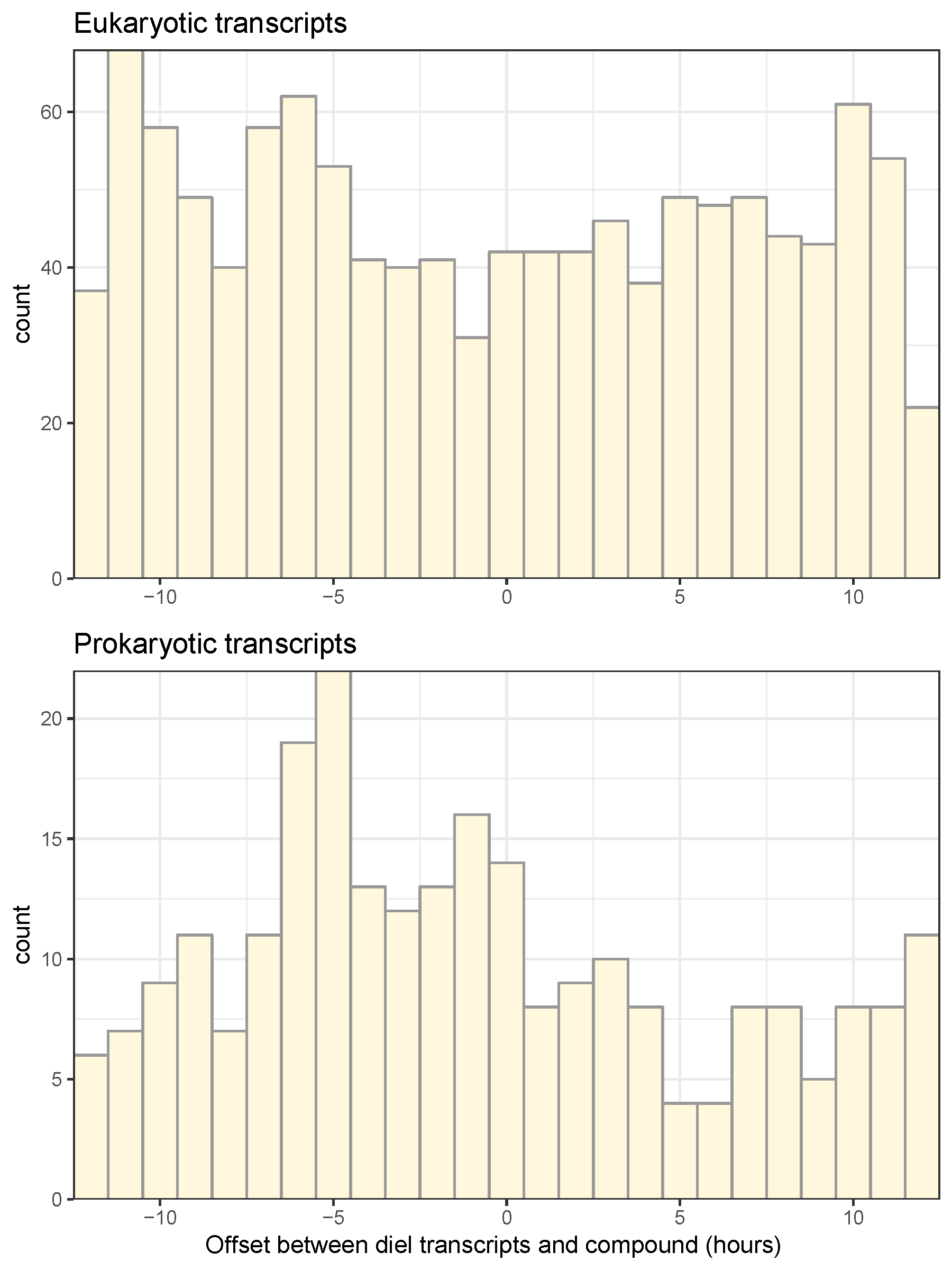

Supplement: FIG S3 [file msystems.00896-20-sf003.tif]

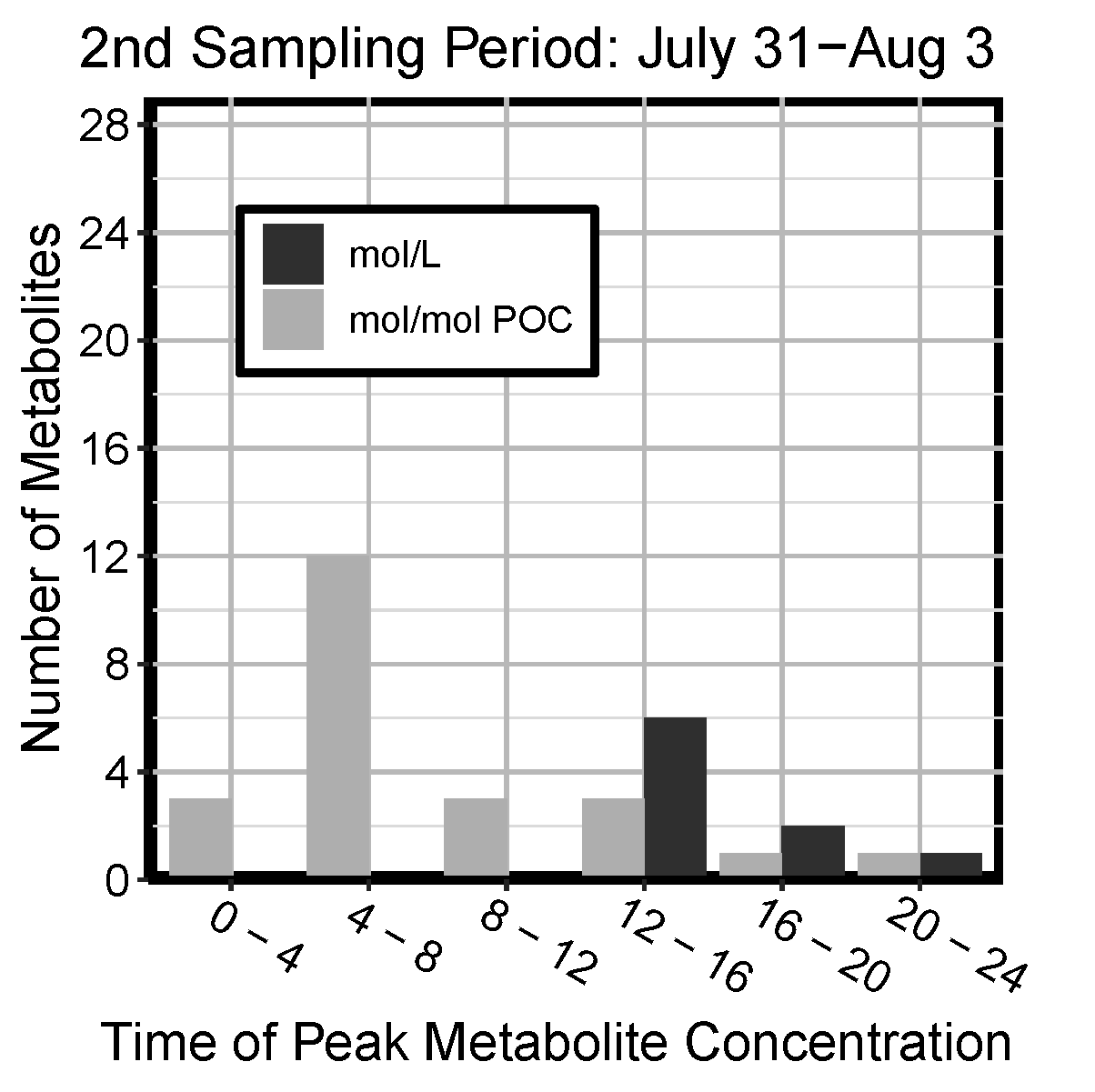

Supplement: FIG S4 [file msystems.00896-20-sf004.tif]
